# Unambiguous Forward-Looking SAR Imaging on HSV-R Using Frequency Diverse Array

**DOI:** 10.3390/s20041169

**Published:** 2020-02-20

**Authors:** Mengdi Zhang, Guisheng Liao, Xiongpeng He, Shengqi Zhu

**Affiliations:** National Lab of Radar Signal Processing, Xidian University, Xi’an 710071, China; liaogs@xidian.edu.cn (G.L.); xidian_hexp@163.com (X.H.); sqzhu@xidian.edu.cn (S.Z.)

**Keywords:** forward-looking imaging, frequency diverse array (FDA), range ambiguity, left–right ambiguity, synthetic aperture radar (SAR)

## Abstract

It is a challenge to realize wide swath imaging due to the conflict between Doppler ambiguity and range ambiguity for hypersonic vehicle (HSV) radar. In addition, there are many conditions requiring the forward-looking imaging. In a forward-looking synthetic aperture radar (SAR) system, left–right ambiguity arises, since two symmetrical targets have the same Doppler frequency magnitude. After selecting an appropriate pulse repetition frequency (PRF) to avoid Doppler ambiguity, we only need to solve the range ambiguity and left–right ambiguity. To handle these issues, this paper proposes an approach to resolve the range ambiguity and left–right ambiguity using the frequency diverse array (FDA). With the range-angle-dependent property of the transmit steering vector, FDA can distinguish the range ambiguous echoes in the spatial frequency domain. By performing transmit beamforming after range compensation, the echo from the desired range region can be extracted from ambiguous echoes. Then, the back projection (BP) algorithm is used to achieve imaging. Next, the echoes of all channels are processed by two receive beamformers, which are designed for the right and left sides, respectively. With the aforementioned procedures, an unambiguous image can be obtained. Simulation results have verified the effectiveness of the proposed approach.

## 1. Introduction

Hypersonic vehicles (HSV) have attracted much attention in recent years because of their military value. HSV, with a velocity of more than five times the speed of sound, generally fly in near space, which means an altitude between 20 km and 100 km [1]. HSV radar (HSV-R) has been a research hotspot in the field of HSV. Compared with traditional airborne radar, HSV-R has higher velocity and altitude, leading to range ambiguity and Doppler ambiguity. Additionally, its forward-looking mode also causes left–right ambiguity. The key to achieving high quality imaging lies in resolving ambiguity. On the one hand, Doppler ambiguity can be avoided by selecting an appropriate pulse repetition frequency (PRF). On the other hand, the range ambiguous echoes can be distinguished by introducing frequency diverse array (FDA).

FDA [2,3] was first introduced by Antonik in 2006. It has attracted growing attention in recent years. The most important difference between FDA and the traditional phased-array is that the former employs a small frequency increment, while the latter has the same frequency between each transmit array. Differing from the traditional phased-array and multiple-input multiple-output (MIMO) antennas, which provide only angle-dependent transmit beampatterns, FDA is capable of forming angle-range-dependent transmit beampatterns. Consequently, the echoes of different range regions can be completely separated in the frequency domain. The characteristics of angle-range-dependent beampatterns are introduced in [4]. The range-angle-dependent transmit beamforming technique for FDA is proposed in [5]. In [6], FDA is used to suppress deceptive jamming in space-borne synthetic aperture radar (SAR) imaging. Additionally, [7] presents a method to suppress range ambiguous clutter with FDA space-time adaptive processing (STAP). In [8], the unambiguous signal reconstruction approach for SAR imaging using FDA is proposed.

Generally, the traditional SAR works in side-looking mode. However, there are many conditions requiring forward-looking imaging, such as missile precision guidance. As symmetric targets have the same magnitude of frequency, a forward-looking SAR system faces left–right ambiguity. In addition, the ground targets distributed in front of the flight trajectory have a small Doppler change rate, which will result in low azimuth resolution. Obtaining high resolution is another problem for forward-looking SAR. However, in bistatic SAR (BiSAR), the transmitter and the receiver are mounted on separate platforms, which makes it possible to improve the above issues. A new imaging algorithm for forward-looking bistatic SAR is proposed in [9]. Double antenna forward-looking SAR imaging is introduced in [10], which can solve left–right ambiguity by utilizing the wave path difference between two channels.

In this paper, we introduce an approach to imaging without ambiguity for forward-looking FDA-SAR. The range ambiguity can be solved by applying a range compensation function and transmit beamformers. Then, the range ambiguous echoes can be separated. Next, back projection (BP) is applied to imaging. Finally, the imaging results of all channels are processed by receive beamforming. As a result, unambiguous imaging for the forward-looking FDA-SAR can be achieved.

The remaining sections are organized as follows. In Section 2, the signal model of the forward-looking FDA-SAR system is presented. In Section 3, a range ambiguity resolution approach is proposed. Section 4 gives a method to distinguish left–right ambiguity. Section 5 discusses the selection of system parameters. In Section 6, some simulations are presented to verify the effectiveness of the proposed method. In the end, conclusions are drawn in Section 7.

## 2. Signal Model

**Figure 1:**
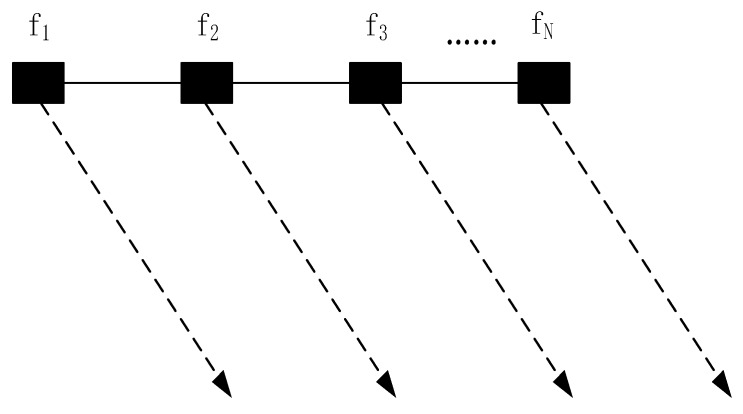
Illustration of frequency diverse array (FDA) with an identical frequency increment.

In the traditional phased array radar, it is assumed that the same carrier frequency is radiated by each array element. Differing from a traditional phased array, the carrier frequency of FDA radiated from each element is identical, with a frequency offset Δf, as shown in Figure 1.

The carrier frequency at the nth transmit antenna can be written as

(1) $$f_{n}=f_{0}+(n-1)\Delta f,\;n=1,2,\ldots ,N,$$

where $f_{0}$ denotes the carrier frequency of the reference antenna. Usually, Δf is far smaller than $f_{0}$ and the system bandwidth B.

The transmit signal of the nth element can be represented as

(2) $$s_{n}(t)=\varphi_{n}(t)\exp(j2\pi f_{n}t)\;,\;n=1,2,\ldots ,N,$$

where $\varphi_{n}(t)$ is the unit energy transmit waveform of the nth antenna. Additionally, it is assumed that waveforms are orthogonal, which can be written as

(3) $$\int_{-\infty }^{+\infty }\varphi_{n}(t){\varphi_{n}}^{*}(t)dt=1,\;n=1,2,\ldots ,N,$$

(4) $$\int_{-\infty }^{+\infty }\varphi_{n}(t){\varphi_{m}}^{*}(t-\tau )\exp[j2\pi \Delta f(n-m)t]dt=0\;m\neq n,\forall \tau ,$$

where the superscript ${}^{*}$ denotes the conjugate operator.

**Figure 2:**
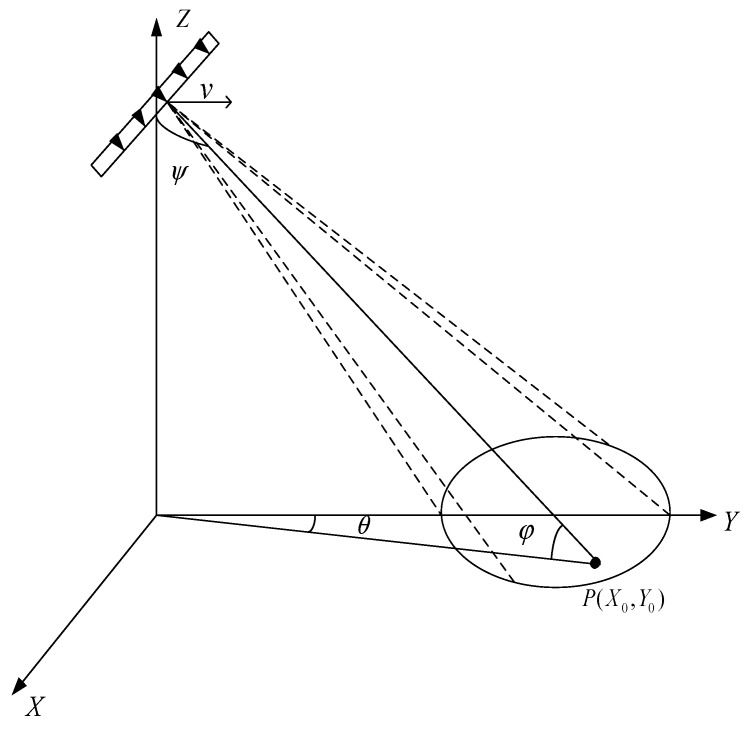
Geometry of the frequency diverse array synthetic aperture radar (FDA-SAR).

Here, we consider a far-field point target at $P(X_{0},R_{0})$, as shown in Figure 2. The echo received by the *m*th element can be expressed as

(5) $$s_{m}(t_{r},t_{a})=\sum_{n=1}^{N}\xi \varphi_{n}(t_{r}-\tau_{mn}(t_{a}))\exp[j2\pi (f_{n}+f_{d})t_{r}]\times w_{a}(t_{a}-\eta_{mn0})\exp[-j2\pi f_{n}\tau_{mn}(t_{a})],$$

where ξ is the complex amplitude of the target echo; $t_{r}$ and $t_{a}$ denote the fast time and the slow time, respectively; $f_{d}$ is the Doppler frequency; $w_{a}(t_{a})$ denotes the azimuth envelope; $\eta_{mn0}$ denotes the instant when the target is closest to the equivalent phase center (EPC); $\tau_{mn}(t_{a})$ is the time delay of the signal transmitted by the *n*th transmit element and received by the *m*th receive element, which can be expressed as

(6) $$\tau_{mn}(t_{a})=\frac{R_{n}(t_{a})+R_{m}(t_{a})}{c},$$

where $R_{n}(t_{a})$ is the slant range between the *n*th transmit element and the target, and $R_{m}(t_{a})$ is the slant range between the *m*th element and the target. They can be expressed as

(7) $$R_{n}(t_{a})=\sqrt{{\left(X_{0}-d_{Tn}\right)}^{2}+{\left(Y_{0}-vt_{a}\right)}^{2}+H^{2}},$$

(8) $$R_{m}(t_{a})=\sqrt{{\left(X_{0}-d_{Rm}\right)}^{2}+{\left(Y_{0}-vt_{a}\right)}^{2}+H^{2}},$$

(9) $$R_{0}=\sqrt{{X_{0}}^{2}+{Y_{0}}^{2}+H^{2}},$$

where *c* is the velocity of light; $d_{Tn}$ and $d_{Rm}$ denote the range of the nth transmit element and the *m*th receive element relative to the reference element, respectively; *v* is the velocity of the platform.

We assume that the scene satisfies the far-field and narrowband assumptions. Then, $\tau_{mn}(t_{a})$ can be expressed as

(10) $$\tau_{mn}(t_{a})=\frac{2[R_{ref}-d_{i}\sin\theta (t_{a})\cos\varphi (t_{a})]}{c},$$

where $R_{ref}$ is the slant range between the target and the reference element, and $\theta (t_{a})$ and $\varphi (t_{a})$ denote the instant azimuth and pitch angle, respectively, as shown in Figure 2.

Here, $d_{i}$ is the range between the *i*th EPC and the reference EPC. It can be written as

(11) $$d_{i}=\frac{(m-1)d_{M}+(n-1)d_{N}}{2},$$

where $d_{M}$ and $d_{N}$ denote the distance between the receive elements and transmit elements, respectively.

The received signal is fed into a bank of matched filters $h_{n}(t)=\varphi_{n}(t)\exp\left(-j2\pi \Delta f_{n}t\right),\;n=1,2,\ldots N$ to separate it from the transmit signal. Then, we can extract the signal transmitted by the *n*th element and received by the *m*th element.

(12) $$s_{mn}(t_{r},t_{a})=\xi {w_{r}}^{n}[t_{r}-\tau_{mn}(t_{a})]w_{a}(t_{a}-\eta_{mn0})\exp[-j4\pi (f_{0}+(n-1)\Delta f)\frac{\left(R_{ref}-d_{i}\sin\theta \cos\varphi \right)}{c}],$$

where ${w_{r}}^{n}(t_{r}-\tau_{mn})=\int_{-\infty }^{+\infty }\varphi_{n}(t_{r}){\varphi_{n}}^{*}(t_{r}-\tau_{mn})\exp(-j2\pi f_{d}(t_{r}-\tau_{mn}))dt$ is the range envelope yielded by the nth filter.

The output signal of the *m*th receive channel can be vectorized as

(13) $$s_{m}=\left[\begin{array}{c} 1\\ \exp\left(-j2\pi \left(\frac{2\Delta f}{c}R-\frac{d_{N}}{\lambda }\sin\theta \cos\varphi \right)\right)\\ \vdots \\ \exp\left(-j2\pi (n-1)\left(\frac{2\Delta f}{c}R-\frac{d_{N}}{\lambda }\sin\theta \cos\varphi \right)\right)\\ \vdots \\ \exp\left(-j2\pi (N-1)\left(\frac{2\Delta f}{c}R-\frac{d_{N}}{\lambda }\sin\theta \cos\varphi \right)\right) \end{array}\right]\times \exp\left[j2\pi (m-1)\frac{d_{M}}{\lambda }\sin\theta \cos\varphi \right],$$

We define the transmit frequency and receive frequency as

(14) $$f_{T}(\psi ,R)=-\frac{2\Delta f}{c}R+\frac{d_{N}}{\lambda }\cos\psi ,$$

(15) $$f_{R}(\psi )=\frac{d_{M}}{\lambda }\cos\psi ,$$

The snapshot vector of the target echo can be written as

(16) $$\begin{array}{ll} s & ={\left[{s_{1}}^{T}\;{s_{2}}^{T}\;\ldots \;{s_{M}}^{T}\right]}^{T}\\ & =\xi b(\psi )⊗a(R,\psi ), \end{array}$$

where ${}^{T}$ denotes the transpose operator; cosψ=sinθcosφ, b(ψ) and a(R,ψ) denote the receive steering vector and the transmit steering vector, respectively.

(17) $$b(\psi )={\left[\begin{array}{cccc} 1 & \exp\left(j2\pi f_{R}\right) & \ldots & \exp\left(j2\pi \left(M-1\right)f_{R}\right) \end{array}\right]}^{T},$$

(18) $$a(R,\psi )={\left[\begin{array}{cccc} 1 & \exp\left(j2\pi f_{T}\right) & \ldots & \exp\left(j2\pi \left(N-1\right)f_{T}\right) \end{array}\right]}^{T},$$

The receive steering vector of FDA is the same as that of the traditional multi-channel SAR system. The difference is that the transmit steering vector of FDA is range-dependent, which can be utilized to separate the range ambiguous echoes.

**Figure 3:**
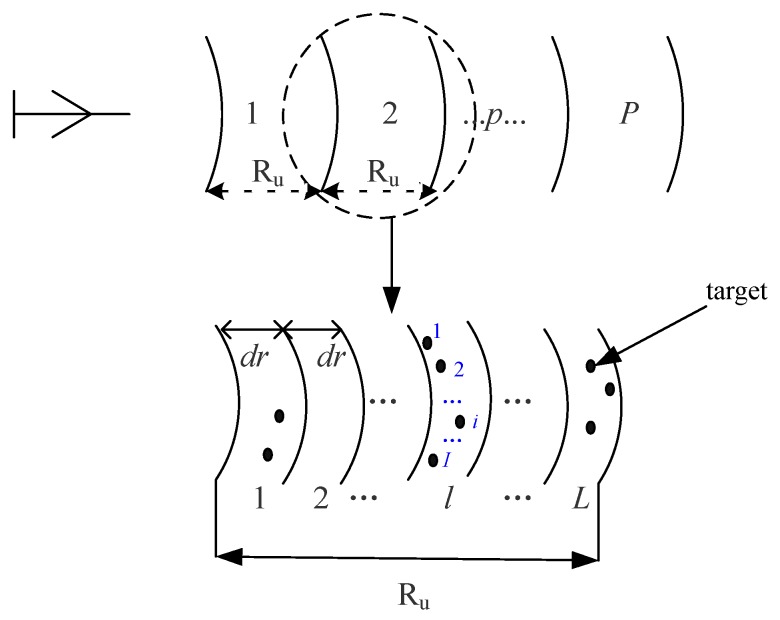
Division of range region.

Compared with the traditional airborne radar, HSV-R has a higher altitude, which results in range ambiguity. The scene can be divided into several range regions according to the maximum unambiguous range $R_{u}=c/2prf$. A range region can be divided into several range bins according to range resolution dr, as shown in Figure 3. The echo steering vector with range ambiguity is given as

(19) $$s_{l}(k)=\sum_{p=1}^{P}\sum_{i}\xi_{p,l,i}b(\psi_{p,l,i}(k))⊗a(\psi_{p,l,i}(k),R_{p,l}(k)),$$

where *i* denotes the index of the targets in a range bin, *l* is the index of range bins, *p* denotes the index of range region, and *k* is the index of the pulse number. The instant slant range between the *i*th target and the reference element can be expressed as

(20) $$R_{p,l}=(p-1)R_{u}+R_{l},$$

## 3. Range Ambiguity Resolution

This part presents an approach to range ambiguity resolution. For HSV-R, we can avoid Doppler ambiguity by choosing a higher PRF. It is assumed that the echo is free of Doppler ambiguity by selecting an appropriate PRF.

It can be seen from Equation (16) that the transmit steering vector is a function of range and angle, a(R,ψ) can be expressed as

(21) a(R,ψ)=c(R)⊙d(ψ),

where ⊙ is the Hadamard product, and c(R) and d(ψ) denote the range and angle steering vectors, respectively. They can be written as

(22) $$c(R)={\left[\begin{array}{cccc} 1 & \exp\left(-j4\pi \frac{\Delta f}{c}R\right) & \ldots & \exp\left(-j4\pi \left(N-1\right)\frac{\Delta f}{c}R\right) \end{array}\right]}^{T},$$

(23) $$d(\psi )={\left[\begin{array}{cccc} 1 & \exp\left(j2\pi \frac{d_{N}}{\lambda }\cos\psi \right) & \ldots & \exp\left(j2\pi \left(N-1\right)\frac{d_{N}}{\lambda }\cos\psi \right) \end{array}\right]}^{T},$$

If we substitute Equation (20) into Equation (22), we have

(24) $$c_{n}(R)=\exp[-j4\pi (n-1)\frac{\Delta f}{c}R_{l}]\exp[-j4\pi (n-1)(p-1)\frac{\Delta f}{c}R_{u}],$$

where $c_{n}(R)$ is the transmit steering vector for the nth transmit element. It can be seen from Equation (24) that the second term of each range region is different. Therefore, we can separate the ambiguous echoes by utilizing the second term. The first term can be compensated by the following compensation function.

(25) $$h_{n}(R_{l})=\exp[j4\pi (n-1)\frac{\Delta f}{c}R_{l}],$$

The compensation steering vector can be written as

(26) $$h_{c}={\left[h_{1}(R_{l})h_{2}(R_{l})\;\ldots \;h_{N}(R_{l})\right]}^{T},$$

Applying the compensation steering vector $h_{c}$ to the range steering vector, we have

(27) $$\begin{array}{ll} {\tilde{c}}_{R,p} & =c(R)⊙h_{c}\\ & ={\left[\begin{array}{cccc} 1 & \exp\left(-j4\pi \left(p-1\right)\frac{\Delta f}{c}R_{u}\right) & \ldots & \exp\left(-j4\pi \left(N-1\right)\left(p-1\right)\frac{\Delta f}{c}R_{u}\right) \end{array}\right]}^{T}, \end{array}$$

After compensation, the transmit frequency can be written as

(28) $${\tilde{f}}_{T}=-2(p-1)\frac{\Delta f}{c}R_{u}+\frac{d_{N}}{\lambda }\cos\psi ,$$

The echo signal after compensation can be expressed as

(29) $${\tilde{s}}_{l}(k)=\sum_{p=1}^{P}\sum_{i}\xi_{p,l,i}b(\psi_{p,l,i}(k))⊗\tilde{a}(\psi_{p,l,i}(k),R_{p,l,i}(k)),$$

where $\tilde{a}(\psi ,R_{p})$ is the transmit vector after compensation, which it can be expressed as

(30) $$\tilde{a}(\psi ,R_{p})={\left[\begin{array}{cccc} 1 & \exp\left(j2\pi {\tilde{f}}_{T}\right) & \ldots & \exp\left(j2\pi \left(N-1\right){\tilde{f}}_{T}\right) \end{array}\right]}^{T},$$

In order to extract the unambiguous echo of each range region from ambiguous echoes, we need to construct a series of weight vectors that can enhance the signal of the desired range region and suppress the ambiguous signal from other range regions. The constraint condition in designing this weight vector can be expressed as

(31) $$\left\{ \begin{array}{l} \underset{w_{p}}{\min}{w_{p}}^{H}R_{s}w_{p}\\ w_{p}\tilde{a}(\psi_{0},R_{p})=1 \end{array}\right.\;,p=1,2,\ldots P,$$

where $w_{p}$ is the weight vector for the *p*th range region, p is the index of the desired range region, and $R_{s}$ is the covariance matrix of the signal.

By solving the constraint condition in Equation (31), we can get the weight vector.

(32) $$w_{p}=\frac{{R_{s}}^{-1}\tilde{a}(\psi_{0},R_{p})}{{\tilde{a}}^{H}(\psi_{0},R_{p}){R_{s}}^{-1}\tilde{a}(\psi_{0},R_{p})},$$

If the system parameters are selected properly according to Section 5, the transmit weight vector can be written as

(33) $$w_{p}=\tilde{a}(\psi_{0},R_{p}),$$

Applying the transmit weight vector $w_{p}$ to the echo after compensation ${\tilde{x}}_{l}(k)$, we have the received signal of the *p*th range region as

(34) $$s_{pl}(k)={\left(w_{p}⊗I_{M}\right)}^{H}{\tilde{s}}_{l}(k),$$

where $I_{M}$ is an M×M identity matrix. After transmit beamforming, the target echo with range ambiguity can be separated. The unambiguous echo can be reconstructed as

(35) $$s=[s_{1}{\;s}_{2}\ldots s_{P}]\;,$$

Afterwards, the signal of the full swath can be obtained.

## 4. SAR Imaging and Left–Right Ambiguity Resolution

Since two symmetrical targets have the same magnitude of Doppler frequency, there is the problem of left and right ambiguity for the forward-looking FDA-SAR system. In this section, an approach to distinguishing left–right ambiguity is proposed. Suppose the range ambiguity echoes have been separated by the method given in Section 3.

After range compression, the echo received by the *m*th channel can be expressed as

(36) $$s_{rm}(t_{r},t_{a})\;=\sigma \sin c[B\pi (t_{r}-\frac{2R_{ref}(t_{a})}{c})\exp[-j\frac{4\pi }{\lambda }R_{ref}(t_{a})]\exp[j2\pi f_{0}(m-1)\frac{d_{m}\sin\theta \cos\varphi }{c}],$$

where B is the bandwidth of the transmitting signal, σ denotes the amplitude of the target echo, and $R_{ref}(t_{a})$ denotes the slant range between the reference element and the target.

Then, the BP algorithm based on pixel reconstruction is applied to the image, which can be written as

(37) $$f_{m}(\rho ,\theta )=\sum \left\{s_{rm}(t_{r},t_{a})\;\exp[j\frac{4\pi }{\lambda }R_{ref}(t_{a})]\right\},$$

where $f_{m}(\rho ,\theta )$ represents the energy of the pixel (ρ,θ) (ρ,θ) received by the *m*th channel, ρ is the slant range between the pixel and the reference element, and θ is the azimuth angle of the pixel.

**Figure 4:**
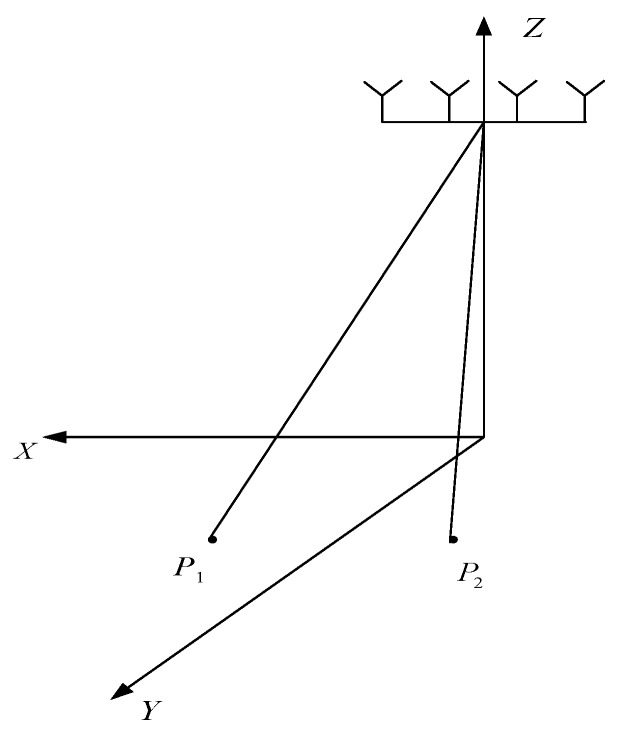
Geometry of the forward-looking SAR.

Since two targets, which are symmetrical about the Y axis (as Figure 4 shows), have the same Doppler frequency magnitude, the two symmetrical pixels accumulate the same energy.

(38) $$\begin{array}{ll} f_{m}(\rho ,\theta ) & =f_{m}(\rho ,-\theta )\\ & =\left[\begin{array}{cc} \exp\left(j2\pi \frac{(m-1)d_{M}\sin\theta \cos\varphi }{\lambda }\right) & \exp\left(j2\pi \frac{(m-1)d_{M}\sin(-\theta )\cos\varphi }{\lambda }\right) \end{array}\right]\times \left[\begin{array}{c} \sigma_{1}\\ \sigma_{2} \end{array}\right], \end{array}$$

where $\sigma_{1}$ and $\sigma_{2}$ are the amplitude of the echo from pixels (ρ,θ) and (ρ,−θ), respectively. The signal received by all receive channels can be expressed as

(39) $$\begin{array}{ll} F(\rho ,\theta ) & =F(\rho ,-\theta )\\ & =\left[\begin{array}{cc} V_{\theta } & V_{-\theta } \end{array}\right]\cdot \sigma , \end{array}$$

where F(ρ,θ) and F(ρ,−θ) are the energy matrix of pixels (ρ,θ) and (ρ,−θ) received by all elements, respectively; σ is the amplitude matrix; $V_{\theta }$ and $V_{-\theta }$ are the array steering vectors of pixels and (ρ,−θ), respectively, which they can be expressed as

(40) $$V_{\theta }={\left[\begin{array}{cccc} 1 & \exp\left(j2\pi \frac{d_{M}\sin\theta \cos\varphi }{\lambda }\right) & \ldots & \exp\left(j2\pi \frac{(M-1)d_{M}\sin\theta \cos\varphi }{\lambda }\right) \end{array}\right]}^{T},$$

and

(41) $$V_{-\theta }={\left[\begin{array}{cccc} 1 & \exp\left(j2\pi \frac{d_{M}\sin\left(-\theta \right)\cos\varphi }{\lambda }\right) & \ldots & \exp\left(j2\pi \frac{(M-1)d_{M}\sin\left(-\theta \right)\cos\varphi }{\lambda }\right) \end{array}\right]}^{T},$$

To resolve the left and right ambiguity, we need to design two filters, so that one can enhance the echo signal of the left area and the other is able to enhance the echo of the right area. Under this constraint, the optimal beamforming problem in the sense of minimum output power for the system can be expressed as

(42) $$\left\{ \begin{array}{l} \underset{w_{1}}{\min}{w_{1}}^{H}Rw_{1}\\ {w_{1}}^{H}\left[\begin{array}{cc} V_{\theta } & V_{-\theta } \end{array}\right]={e_{1}}^{T} \end{array}\right.\;,$$

and

(43) $$\left\{ \begin{array}{l} \underset{w_{2}}{\min}{w_{2}}^{H}Rw_{2}\\ {w_{2}}^{H}\left[\begin{array}{cc} V_{\theta } & V_{-\theta } \end{array}\right]={e_{2}}^{T} \end{array}\right.,$$

where $w_{1}$ and $w_{2}$ represent the weight that can enhance the echo from the left and the right, respectively. Here, R is the covariance matrix of the signal and can be written as

(44) $$R=F(\rho ,\theta )F{\left(\rho ,\theta \right)}^{H},$$

where $e_{1}$ is the first column of the two-dimensional identity matrix; $e_{2}$ is the second column of the two-dimensional identity matrix; $w_{1}$ and $w_{2}$ can be given by

(45) $$w_{1}=\frac{R^{-1}V_{\theta }}{V_{\theta }R^{-1}V_{\theta }}e_{1},$$

and

(46) $$w_{2}=\frac{R^{-1}V_{-\theta }}{V_{-\theta }R^{-1}V_{-\theta }}e_{2},$$

Applying the obtained receive weight $w_{1}$ and $w_{2}$ to the received signal after BP, we can separate the signal from the pixels (ρ,θ) and (ρ,−θ), which can be expressed as

(47) $$F_{L}={w_{1}}^{H}F,$$

(48) $$F_{R}={w_{2}}^{H}F,$$

By performing receive beamforming for all pixels, we can obtain the image without left–right ambiguity.

**Figure 5:**
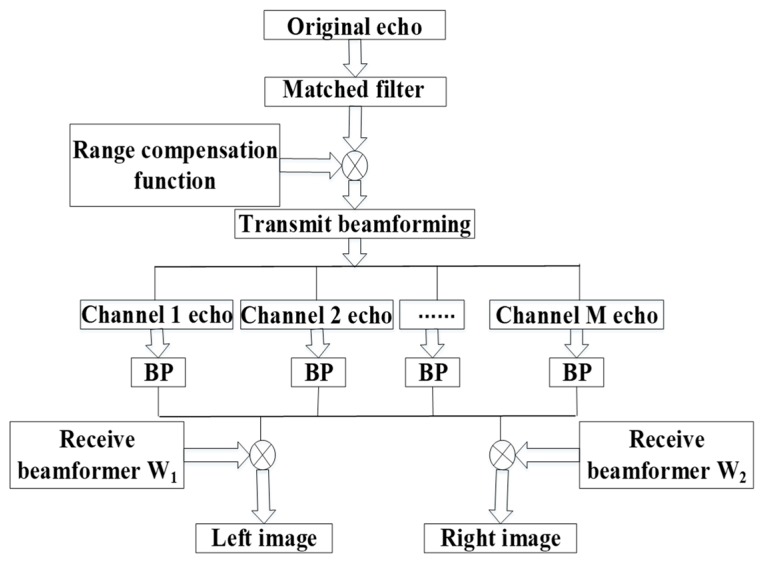
Processing flow chart of the forward-looking FDA-SAR. Note: BP, back projection.

The processing flow chart can be summarized as in Figure 5. After matched filtering, the signal is processed by range compensation and transmit beamforming to resolve the range ambiguous echoes into several unambiguous parts. Then, by performing BP on the echoes without range ambiguity received by each channel, we will have the image with left and right ambiguity. Next, the echoes after performing BP are processed by two receive beamformers, respectively. Finally, we can obtain the unambiguous images of the two sides.

## 5. Selection of System Parameters

To achieve optimal range ambiguity resolution performance, the system parameter design for the forward-looking FDA-SAR is discussed in this section. In order to analyze the suppression effectiveness of the echo from the undesired range region, a function is defined by

(49) $$\begin{array}{ll} f(q) & =\left|{w_{p}}^{H}a(\psi ,R_{uq})\right|\\ & =\left|\frac{\sin[2\pi N(p-q)\frac{\Delta f}{c}R_{u}]}{\sin[2\pi (p-q)\frac{\Delta f}{c}R_{u}]}\right|, \end{array}$$

where q is the index of the range region. In order to suppress the echo from the undesired range region, we need q=p to be the only maximizer of f(q). Therefore, the condition that the frequency increment Δf needs to satisfy can be expressed as

(50) $$\frac{2\Delta f}{c}R_{u}=k,$$

where k is an irrational number. Substituting $R_{u}=\frac{c}{2prf}$ into Equation (50), we have

(51) $$\frac{\Delta f}{prf}=k,$$

The spatial frequency of the transmit steering vector after compensation can be written as

(52) $$\begin{array}{ll} {\tilde{f}}_{T} & =\frac{d}{\lambda }\cos\psi -\frac{2\Delta f}{c}R_{u}(p-1)\\ & =\frac{d}{\lambda }\cos\psi -k(p-1), \end{array}$$

To extract the echo from different range regions, some considerations are given. On the one hand, the spatial frequency of different range regions cannot overlap, which means $k\geq B_{s}$. On the other hand, in order to avoid Doppler ambiguity, the spatial frequency of all range regions must be within [−0.5,0.5], which requires $kN_{a}\leq 1$. In addition, the difference of the spatial frequency between adjacent regions must be maximized, which ensures that the echoes of different range regions can be separated easily. All of the above requirements can be expressed as

(53) $$\left\{ \begin{array}{l} \max\;k\\ s.t.\;k\geq B_{s}\\ \;\mathrm{kNa}\leq 1 \end{array}\right.,$$

Then, we have

(54) $$k_{\max}=\frac{1}{N_{a}},N_{a}<\frac{1}{B_{s}},$$

Moreover, the maximum range ambiguity number is

(55) $$N_{a\max}=\min[N,\frac{1}{B_{s}}],$$

## 6. Simulation

In this section, the imaging results of the forward-looking FDA-SAR are given to validate the proposed imaging algorithm. In the simulation, the FDA-SAR system parameters and the position information of targets are listed in Table 1 and Table 2, respectively. The center of region 1 is taken as the scene center.

**Figure 6:**
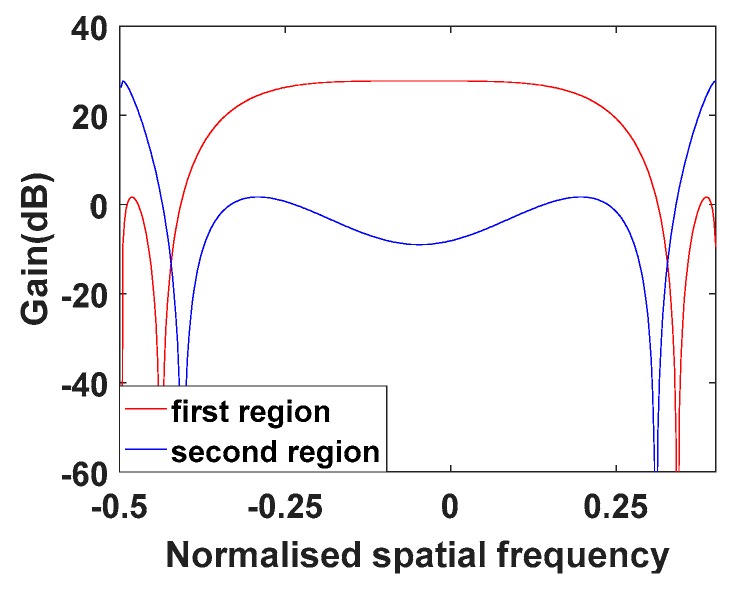
Filter response of the transmit beamformer designed for the first region.

Figure 6 shows the filter response of the transmit beamformer designed for first region. As can be seen, the range ambiguous echoes can be separated in the spatial frequency.

**Figure 7:**
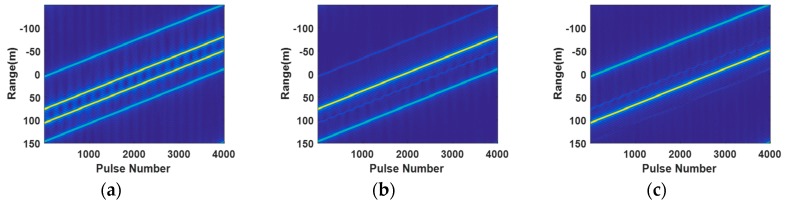
Signal after range compression: (**a**) Original signal after range compression. (**b**) Signal from the first range region after range ambiguity resolution and range compression. (**c**) Signal from the second range region after range ambiguity resolution and range compression.

**Figure 8:**
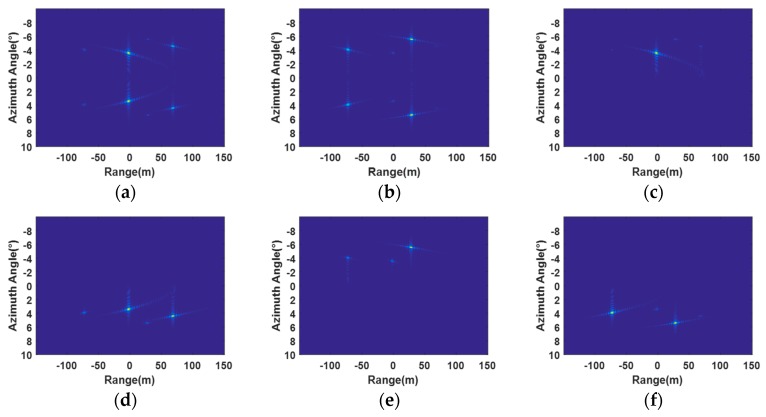
Imaging results of the forward-looking FDA-SAR. (**a**) Imaging of the first range region by BP. (**b**) Imaging of the second range region by BP. (**c**) Left side of the first region after receive beamforming. (**d**) Right side of the first region after receive beamforming. (**e**) Left side of the second range region after receive beamforming. (**f**) Right side of the second range region after receive beamforming.

The imaging of the targets by the proposed method for the forward-looking FDA SAR is presented in Figure 7 and Figure 8. The trajectory of the targets after range compression is given in Figure 7a. Due to left/right ambiguity, the trajectories of target 1 and target 2 are overlapped. The trajectories of target 4 and target 5 are also overlapped. The echoes of the first range region after extracting from the range ambiguous echoes by compensation and transmit beamforming are shown in Figure 7b. It can be seen that the range ambiguous energy of target 4, target 5 and target 6 has been well suppressed by applying transmit beamforming. The echoes of the second range region are presented in Figure 7c. We can observe that the energy of target 1, target 2 and target 3 is also well suppressed.

The imaging of the targets in the first and the second range regions, which are focused by BP, are shown in Figure 8a,b, respectively. It is apparent that the proposed range ambiguity resolution approach has effectively resolved the range ambiguous echoes into two unambiguous parts. In Figure 8a, it can be seen that the energy of the targets in the first range region is well focused, while that in the second range region is suppressed. In addition, the energy of target 1, target 2, and target 3 is distributed on both the left and right sides of the scene because of left–right ambiguity. In Figure 8b, it can be observed that the energy from the second range region is focused by BP. Additionally, the energy of target 4, target 5, and target 6 is distributed on both the left and right sides of the scene. By applying receive beamformers to the images after BP, we can resolve the left–right ambiguous images into two unambiguous parts. Figure 8c,d show the imaging of the targets from the first range on the left and right sides of the scene, respectively. Compared with Figure 8a, it can be observed that the energy from target 1 is well focused, as shown in Figure 8c, by applying the receive beamformer designed for the left side of the scene. We can observe that the targets on the right side of the scene from the first range region are well focused in Figure 8d by applying the receive beamformer designed for the right side of the scene. Figure 8e,f show the imaging of the targets from the second range on the left and right sides by applying receive beamforming, respectively. From Figure 8c–f, it can be concluded that the proposed left–right ambiguity resolution approach has effectively extracted the targets in the desired side of the scene from left and right ambiguous echoes.

The peak side lobe ratio (PSLR) of targets and the integral side lobe ratio (ISLR) are listed in Table 3. The resolution of the images are listed in Table 4. It is obvious that target 1, target 2, target 3, target 4, target 5, and target 6 are all well focused. The computational time of the algorithm is $O(N_{r}N_{m}(2+N_{\theta }M)))$(Nr is the number of range bins, $N_{m}$ is the number of pulses, $N_{\theta }$ is the number of azimuth bins, while *M* is the receive elements number).

**Figure 9:**
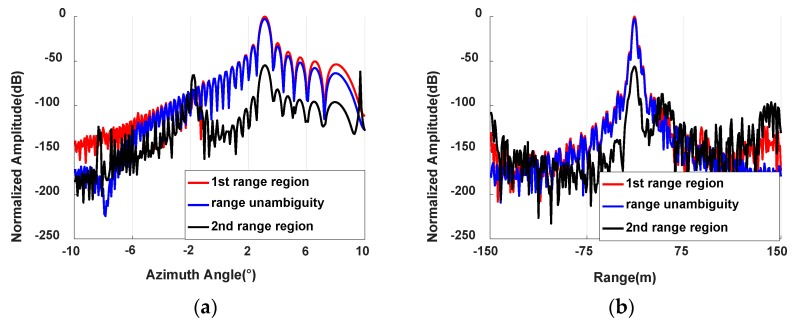
Amplitude comparison. (**a**) Amplitude comparison at the 150th range gate. (**b**) Amplitude comparison at the 164th azimuth gate.

Figure 9a,b show the amplitude of the 150th range gate and the amplitude of the 164th azimuth gate, respectively. Target 1 is in the 150th gate and the 164th azimuth gate of the first range region. Thus, the actual energy of target 1 should be in the first range region. However, the energy of target 1 is distributed in the first and the second range regions due to range ambiguity. The blue line represents the amplitude of target 1 without range ambiguity. The red line and black line denote the amplitude of the first range region and the second range region after performing range ambiguity resolution, respectively. As can be seen from Figure 9, there is no obvious difference between the red line and the blue line. Furthermore, the peak of the black line is lower than that of the red line and the blue line, and the gap is greater than 50 dB. This shows that the range ambiguity resolution proposed in this paper can effectively suppress the range ambiguous echoes.

**Figure 10:**
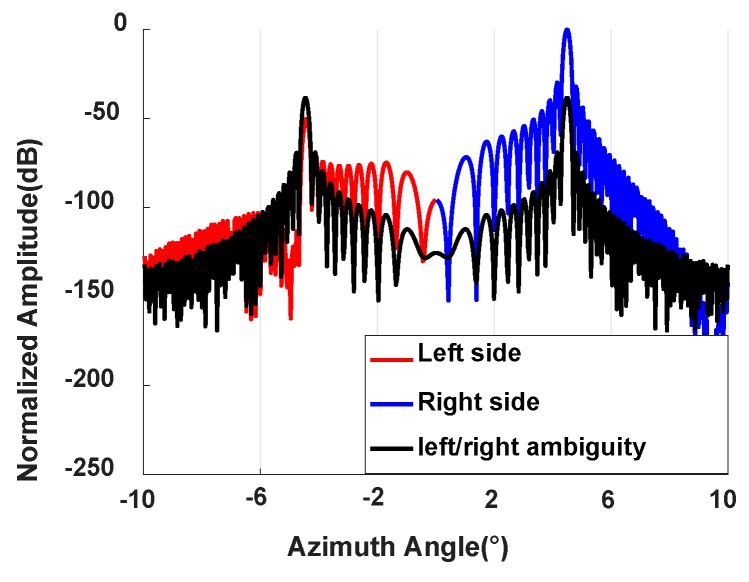
Amplitude comparison at the 220th range gate in the first range region.

Figure 10 shows the amplitude of the 220th range gate in the first range region after performing BP. The black line represents the amplitude of the 220th range gate in channel 1 after BP. Target 3 is in the 220th range gate. It can be seen from the black line that the energy of target 3 is on both sides before left–right ambiguity resolution. The red line and the blue line denote the amplitude of the 220th range gate on the left side and the right side, respectively, after left–right ambiguity resolution. The actual energy of target 3 should be distributed on the right side. It can be seen from Figure 10 that the peak of the red line is lower than that of the blue line, and the gap is greater than 50 dB. This shows that the left–right ambiguity resolution proposed in this paper can effectively suppress the left–right ambiguous echoes.

## 7. Conclusions

In this paper, an approach to range ambiguity and left–right ambiguity resolution for HSV forward-looking FDA-SAR is proposed. Utilizing the proposed algorithm, high-resolution and wide-swath (HRWS) imaging can be achieved. Compared with the conventional forward-looking SAR imaging algorithm, the algorithm presented in this paper can distinguish the range ambiguous echoes by FDA. FDA introduces the wave–path difference among the elements, leading to the range-angle-dependent property of the transmit steering vector. Therefore, FDA is capable of separating the range ambiguous echoes in the spatial frequency domain.By performing range compensation and transmit beamforming on the echoes after matched filtering, the range ambiguous echoes can be resolved into several parts without range ambiguity. Afterwards, the BP algorithm is applied to the received data of each channel, allowing the images with left–right ambiguity to be achieved. By exploiting the receive degree-of-freedom in space, left–right ambiguous echoes can be resolved into two parts. Subsequently, we can obtain the HRWS imaging. Evidently, the simulation results have verified the effectiveness of the proposed range ambiguity and left–right ambiguity resolution approach for the forward-looking FDA-SAR system. For future work, we will explore the waveform design issues for the FDA-SAR system.

## Figures and Tables

**Table 1:** Forward-looking FDA system parameters. Note: PRF, pulse repetition frequency.

Symbol	Parameter	Value
*f*_0_	Carrier frequency	5.3 GHz
Δf	Frequency increment	21,111 Hz
*N*	Transmit element number	4
*M*	Receive element number	8
*d*	Element space	0.0283 m
*V_a_*	Platform velocity	1700 m/s
*F_r_*	PRF	43,331 Hz
*N_a_*	Range ambiguity number	2
*F_r_*	Fast time sampling frequency	150 MHz
*R_u_*	Maximum unambiguous range	3462 m

**Table 2:** Target position.

Target Number	Range Region	Range to the Scene Center	Azimuth Angle
1	1	0 m	−3.5°
2	1	0 m	3.5°
3	1	70 m	4.5°
4	2	Ru +30 m	−5.5°
5	2	Ru +30 m	5.5°
6	2	Ru −70 m	4°

**Table 3:** Imaging performance of targets. Note: PSLR, peak side lobe ratio; ISLR, integral side lobe ratio.

Target Number	PSLR of Range/dB	PSLR of Azimuth/dB	ISLR of Range/dB	ISLR of Azimuth/dB
1	−12.95	−15.51	−12.55	−14.60
2	−12.94	−15.34	−12.55	−14.62
3	−12.90	−15.68	−11.67	−14.69
4	−13.21	−15.51	−11.36	−14.64
5	−13.17	−15.74	−11.36	−14.68
6	−13.11	−15.42	−11.33	−14.66

**Table 4:** Imaging resolution.

Target Number	IRW of Range/m	Theoretic Range Resolution/m	IRW of Azimuth/°	Theoretic Azimuth Resolution/°
1	1.73	1.5	0.24	0.15
2	1.70	1.5	0.24	0.15
3	1.72	1.5	0.20	0.12
4	1.76	1.5	0.16	0.10
5	1.75	1.5	0.15	0.10
6	1.80	1.5	0.20	0.13
